# Cancer and Ageing Reflections for Elders (CARE): Australian Adaptation of a Psychotherapy for Older Adults With Cancer

**DOI:** 10.1002/pon.70402

**Published:** 2026-02-14

**Authors:** Kirsty Galpin, Sharon He, Joanne Shaw, Haryana Dhillon, Christian Nelson, Lisa Beatty, Michelle Kelly, Mariko Carey, Agatha Conrad, Amelia Bartczak, Brian Kelly

**Affiliations:** ^1^ School of Psychology Psycho‐Oncology Co‐operative Group (PoCoG) Faculty of Science The University of Sydney Sydney NSW Australia; ^2^ Department of Psychiatry and Behavioral Sciences Memorial Sloan Kettering Cancer Centre New York New York USA; ^3^ Flinders University Institute for Mental Health and Wellbeing, College of Education, Psychology and Social Work Flinders University Adelaide SA Australia; ^4^ School of Psychological Science Newcastle University Newcastle NSW Australia; ^5^ School of Medicine and Public Health Newcastle University Newcastle NSW Australia; ^6^ Hunter New England Mental Health Service School of Medicine and Public Health College of Health Medicine and Wellbeing Newcastle University Newcastle NSW Australia

**Keywords:** aged, cancer, cross‐cultural comparison, depression, oncology, psycho‐oncology, telemedicine

## Abstract

**Background:**

Depression is common among older adults with cancer. The USA‐developed CARE (Cancer and Ageing: Reflection for Elders) psychotherapy intervention, specifically addresses the unique needs of older people (≥ 70 years) navigating the challenges of ageing, depression, and cancer.

**Aims:**

To review and tailor the CARE resources to ensure they are culturally appropriate and acceptable for older Australians.

**Methods:**

Semi‐structured cognitive ‘think aloud’ interviews were conducted with older Australians (≥ 70 years) diagnosed with cancer. Participants reviewed the intervention resources for each session providing feedback on content relevance and understandability. Content analysis was used to analyse the interviews.

**Results:**

We completed 20 cognitive interviews. Participants had a mean age of 74 years (range 70–79) and most with a diagnosis of blood (55% *n* = 11) or breast cancer (45%, *n* = 9) within 10 years. Resource content resonated with participants and a telephone‐delivered intervention was acceptable. Participants emphasised the need to simplify wording and modify language to reflect Australian culture. In Australia ‘elders’ is a cultural term used by First Nations peoples to identify a custodian of knowledge; most participants suggested changing this. For some participants, the analogy of ageism to racism used felt unfamiliar.

**Conclusions:**

This study highlights that cultural adaptation of psycho‐oncology interventions is required, even between English‐speaking countries, to ensure cultural appropriateness and enhance feasibility, acceptability and to maximise uptake for older adults facing cancer and depression.

## 1 Background

Sixty percent of cancer diagnoses and 70% of cancer deaths occur in older adults [1]. With an ageing population globally, caring for older people with cancer poses a unique challenge given the presence of multimorbidity and other vulnerabilities. Presence of co‐morbid illnesses is associated with increased psychological morbidity in older adults, therefore those living with cancer represent a uniquely high‐risk group, with estimates of depression as high as 27% [2, 3, 4, 5]. Left untreated, depression can interfere with cancer treatment adherence [2, 6, 7] and is associated with substantial disability, poor recovery and higher risk of suicide [3, 4]. Despite this, older individuals continue to be among the least likely to access mental health services [5, 8]. This is compounded in Australia for the 34% of older adults living outside metropolitan centres [9, 10] who experience more social isolation, mental health stigma, and poorer access to health care [11].

Although there are interventions focussed on treating depression in the general population and in cancer patients separately, there remains a lack of psycho‐oncology interventions designed specifically to treat depression *and* address the specific needs of older cancer patients. In response, Nelson et al. 2019 [12] developed a novel intervention, Cancer and Ageing: Reflections for Elders (CARE) to treat depression in older cancer survivors. The CARE intervention is a manualised, five session telephone‐delivered intervention that (i) integrates Folkman's stress and coping theory [13, 14, 15, 16, 17] and Erikson's developmental stages of life theory [18, 19], expanded on by Vaillant [20]; and, (ii) feedback from older people with cancer. CARE was developed as a telephone intervention to facilitate access for those with limited mobility, financial, and geographic barriers to care.

The CARE intervention is delivered over a 2‐month period and includes four additional, brief, booster sessions, delivered monthly. CARE encompasses reappraisal techniques important for successful ageing, reflective exercises and cognitive restructuring and intervention techniques such as active problem solving, information giving and receiving, discussion of concerns, and social support. A pilot study (*n* = 59) of CARE found the intervention to be feasible and demonstrated a reduction in depression scores (*d* = 0.58 [CI:0.04–1.12], *p* = 0.01). The intervention is currently being evaluated in a US randomised controlled trial (RCT) to assess efficacy.

Implementing evidence‐based interventions across different cultures and healthcare systems is a major challenge in psycho‐oncology. Meta‐analytic reviews in mental health highlight the improved effectiveness of interventions tailored to the local context [21]. Adaptation, a key concept in implementation science, is a process of thoughtful and deliberate modification of an intervention, to improve local fit [22]. Culture is a complex construct and adaptation to meet the needs of a cultural group involves consideration of intervention language, culture and context in such a way that it is compatible with the individual's cultural patterns, meanings, and values [23]. Prior to evaluating the efficacy of the CARE intervention in Australia, the research team used co‐design principles, guided by the FRAME framework for reporting adaptations and modifications to evidence‐based interventions [22] to evaluate acceptability and culturally modify CARE content to improve engagement, acceptability and potential clinical outcomes in the Australian context.

### 1.1 Research Question

The aim of this study was to (i) explore the acceptability of the CARE intervention and (ii) identify adaptations to content to increase local relevance for older Australians with cancer experiencing depression.

## 2 Methods

### 2.1 Design

Qualitative cognitive “think aloud” interviews were conducted [24] and analysed using content analysis, guided by the FRAME framework [22]. Planning, conduct, and reporting followed the 32‐item checklist in Consolidated Criteria for Reporting Qualitative Research (COREQ) guidelines [25].

### 2.2 Participants and Recruitment

Eligible participants were older adults (≥ 70 years) with an experience of cancer (diagnosed ≤ 10 years ago) with access to an internet connection for Zoom videoconferencing. The research team contacted participants through an email expression of interest (EOI) sent to older adults with cancer who had previously participated in studies exploring their cancer experiences and consented to being contacted for future research. Participation was by self‐selection in response to the EOI.

### 2.3 Data Collection

Interested participants were directed to a short screening survey to confirm eligibility. After providing consent participants completed an online survey which included demographic, cancer experience, health seeking behaviour questions and contact details to arrange an interview.

Interview participants were emailed the CARE patient resources. Although each participant only viewed one of the CARE session resources, resources for each CARE session were reviewed by four to five participants. A ‘scene setting’ explanation including the session purpose and session number in the programme was provided.

The cognitive think aloud interviews were semi‐structured in nature. Prompts and probing questions were used as required. During the interviews, participants were asked to “think aloud” as they reviewed the wording, phrases, and examples in the resources and provided feedback on the relevance, acceptability, and understandability. Participants were also asked to provide feedback on the acceptability of the telephone‐based intervention. Interviews were conducted via Zoom by two female members of the research team (KG & SH) both experienced in qualitative research methods. Interviews were recorded and transcribed verbatim. A summary of the resource intervention content is in Supporting Information S1.

### 2.4 Analysis

Participant characteristics were analysed descriptively. Interviews were independently reviewed by two interviewers (KG, SH) and analysed using content analysis following a conventional inductive approach [24] based on the steps outlined by Vears and Gillam (2022) [26] (See Supporting Information S1). Words, phrases and examples participants frequently commented on were identified and organised independently by both interviewers. Concepts and categories were developed across interviews, no pre‐defined theories or codes underpinned the development of categories. Concepts and proposed adaptations to each resource content, with rationales, were presented in summary tables and thematically collated. Data were managed using Excel and Word.

## 3 Results

Twenty‐three participants expressed an interest in participating, and 20 participated in the interviews. Reasons for non‐participation were ineligibility (*n* = 1), incomplete survey responses (*n* = 1) and unavailability for interview (*n* = 1). Median interview length was 59.8 min. Participants had a mean age of 74 years (range 70–79), most were female (65%, *n* = 13), with a diagnosis of blood (55%, *n* = 11) or breast cancer (45%, *n* = 9) within the last 10 years. Six (30%) reported having sought support from a mental health professional. A third (30%) of participants resided in regional and rural/remote areas (See Table 1 for full participant characteristics).

**TABLE 1:** Participant characteristics (*n* = 20).

Characteristic	Mean
Age	74 (range 70–79)

	***n* (%)**
Gender	
Female	13 (65)
Male	7 (35)
Year of diagnosis	
Within last year	1 (5)
2–5 years	12 (60)
6–10 years	7 (35)
ARIA postcode^a^	
Metropolitan	14 (70)
Regional	1 (5)
Rural/remote	5 (25)
Aboriginal or Torres strait islander	
No	19 (95)
Yes, Torres strait islander	1 (5)
Language spoken as child	
English	19 (95)
Other	1 (5)
Language spoken at home	
English	19 (95)
Other	1 (5)
Country of birth	
Australia	14 (70)
Other	6 (30)
Highest level of education	
Year 10 or below or equivalent	1 (5)
Year 12 (HSC) or equivalent	5 (25)
Bachelor's degree	2 (10)
Postgraduate degree	5 (25)
Tafe certificate/diploma	5 (25)
Other (grade certificate/graduate diploma)	2 (10)
Cancer type^b^	
Breast	9 (45)
Blood (leukaemia, lymphoma, myeloma)	11 (55)
Melanoma	1 (5)
Stage of cancer at diagnosis	
Stage 1	3 (15)
Stage 2	2 (10)
Stage 3	4 (20)
Stage 4	4 (20)
Not sure	7 (35)
Current treatment status	
Currently receiving active treatment	4 (20)
Currently receiving maintenance treatment	7 (35)
Treatment is complete	9 (45)
Ever seen a mental health professional	
Yes	6 (30)
No	14 (70)
Currently seeing mental health professional for support related to cancer	
Yes	2 (10)
No	18 (90)
Professional(s)^c^ who participants have gone to for advice or help in past 8 weeks for personal or emotional problems	
Mental health professional (e.g., counsellor, psychologist, psychiatrist, social worker)	2 (10)
Family doctor/general practitioner	5 (25)
Other (friend)	1 (5)
I have not sought help from anyone for my problem	2 (10)
Not applicable	12 (60)

^a^ *ARIA postcode: The Accessibility/Remoteness Index of Australia (ARIA) categorises areas according to their distance from “service centres”. ARIA defines five categories of remoteness and is available for a variety of geographical units including postcodes*.

^b^ *Note: n = 21 as 1 participant reported two cancer diagnoses*.

^c^ *multiple responses allowed*.

### 3.1 Qualitative Analysis

Qualitative analysis identified four themes: (i) Acceptability of telephone‐delivered psychological care; (ii) Defining participant preferences for age and coping; (iii) Relevance of concepts discussed in the CARE intervention; and, (iv) Clarity, relevance and understanding of words and images. A full list of proposed changes with a rationale are provided in Table 2.

**TABLE 2:** Summary of resource changes and rationales.

*Resource #*	*Changes*	*Rationale*
1 (Background & session 1)	Resource text entered into an online health literacy editor (SHeLL editor) to assess grade reading score and ease/understandability. For example: ‘implemented’ to ‘made’ ‘infirmity’ to ‘disability’ ‘burgeoning’ to ‘growing’ Ensure Australian spelling (i.e., humour to humour) Good Aussie language, or it sounds too stiff (P02) Dot points/bullet points to break up text Simplify background study information, Australian data Acronyms/abbreviations expanded ‘Elders’ to ‘older person’ ‘Coping’ to ‘managing’/’adjusting’	Simplify language and words being used, and shorten sentences Ensure understandability/relatability Make language kind and compassionate/less clinical Increase relevance of information presented Elders in Australia refers to First Nations people Coping viewed less positively than alternatives i.e. no plan, not progressing
2 (Session 2)	Resource text entered into an online health literacy editor (SHeLL editor) to assess grade reading score and ease/understandability. For example: ‘Acuity’ to ‘alertness’ Altruism sentence reworded to ‘with kindness and helping’ ‘Adversity’ to ‘hard times’ Ensure Australian spelling Reduce Americanisms i.e., ‘True grit’ changed ‘determination’ ‘Alzheimer's’ to ‘dementia’ ‘Elders’* to ‘older person’ Remove wheelchair picture, replaced with different picture. Activities updated i.e.,’ write to a friend’ to ‘call a friend’ and ‘books on tape’ to ‘audio books’ Pastimes added i.e. included sports, RSL, swimming, going for walk Reduced religious sounding sentences i.e. ‘Fills me with joy’	Simplify language and words being used, and shorten sentences Ensure understandability/relatability Make language kind and compassionate/less clinical Elders in Australia refers to First Nations people, *Replaced Elders with ‘Older’ with the exception of this sentence in this resource which was maintained as context was understandable: Increased ability to manage hard times, called “wisdom” of ‘elders’ Picture of man in wheelchair viewed negatively, participants do not resonate with image Activities and pastimes made more relatable to Australians
3 (Session 3)	Resource text entered into an online health literacy editor (SHeLL editor) to assess grade reading score and ease/understandability. For example: ‘Confidant’ to ‘close friend’ ‘feeling marginalised’ to ‘feeling less understood’ Activities added i.e., sports, coffee with friends, country race meetings and activities removed i.e., ‘ballet’ Remove comparison of racism to ageism Brief explanation of Yogi Berra provided i.e., Yogi Berra (an American professional baseball catcher)	Simplify language and words being used, and shorten sentences Ensure understandability/relatability Make language kind and compassionate/less clinical Activities removed or added to be more relatable to Australians, and those living in rural areas Comparison of ageism to racism was not well understood, not used in Australian context
4 (Session 4)	Resource text entered into an online health literacy editor (SHeLL editor) to assess grade reading score and ease/understandability. For example: ‘amalgam’ to ‘mixture’ ‘societal treasure’ to ‘wealth of knowledge ‘transmit’ to ‘passed on’ ‘Elders’ to ‘older people’ Addition of other examples i.e., “home duties” or “tradesmen” under work life Addition of other examples i.e., ‘part of rotary clubs, bowling clubs'	Simplify language and words being used, and shorten sentences Ensure understandability/relatability Make language kind and compassionate/less clinical Addition of other examples to ensure diversity of work experiences Addition of other examples reflective or more relatable to Australians
5 (Session 5 & Booster sessions)	Resource text entered into an online health literacy editor (SHeLL editor) to assess grade reading score and ease/understandability. For example: ‘techniques’ to ‘strategies’ ‘Elder’ to ‘older’ Remove picture of woman, replaced with different picture. Quote used was replaced with different quote.	Simplify language and words being used, and shorten sentences Ensure understandability/relatability Make language kind and compassionate/less clinical Image of woman was replaced as participants viewed image negatively, was not relatable Quote used was “over the top” (P20), uncommon, and not relatable.

### 3.2 Telephone‐Delivered Psychological Care

A telephone‐delivered psychological intervention was viewed by participants as a practical, acceptable option to overcome access barriers, with many participants expressing comfort with the format, especially for those in rural areas. Participants acknowledged the telephone does not have technology accessibility issues associated with videoconferencing, however many participants reported face‐to‐face interactions afforded a personal connection, and seeing body language, which video can provide, improved understanding.

a phone is probably more comfortable. They could put it on speaker, and they can feel more relaxed with it rather than sort of stressing that something's going to drop out… I think a phone's very acceptable.

(P11)

I think zoom sessions are preferable to just telephone sessions…seeing people…. You don't feel that the sort of detachment to the same extent

(P20)

Overall participants resonated with the intervention content and particularly the need for a specific programme to address the unique needs of older people. Although some participants noted usefulness of the intervention will ultimately be dependent on the healthcare professional delivering and person receiving the intervention.

Depend on the person, how they speak I guess, how they offer the questions.

(P21)

### 3.3 Defining Participant Preferences for Age and Coping

Across sessions, currently ‘*elders*’ is used to indicate older people. While some participants indicated they did not mind the term elders, it was more broadly highlighted that in Australia it is typically reserved for Aboriginal and Torres Strait Islander Peoples. Participants suggested elderly, seniors, or older people as more commonly used references to older Australians. Others perceived ‘*elder*’ or *‘old’* as overused, but didn't mind the connotations, seeing it as a natural reflection of age and accumulated wisdom:

Well, we are elders… I don't find it at all offensive, I think. Yeah, that's what I am. I'm an elder.

(P08)

Similarly, in session 2, the intervention resources use the analogy of racism to describe ageism (i.e., age‐based discrimination). Participants strongly disagreed with the comparison, highlighting the two were very different concepts. Some participants noted that racism felt more harmful than ageism. While acknowledging for some, ageism results in feeling ignored or marginalised, others found it more benign, such as receiving help in public spaces when struggling with tasks like grocery shopping.

It’s [ageism] nothing like racism.

(P09)

The context in which ‘coping’ is used was also highlighted by some participants as important. The use of ‘coping’, for acute changes appeared acceptable, but viewed as unhelpful for ongoing changes and/or within the context of the programs aims. Participants perceived ‘management’ of ageing and cancer as the goal, not merely coping:

…managing is a very solid thing [compared with coping]. You've got a plan. You've got it worked out, and you follow the steps.

(P02)

An alternative word suggested was ‘*adjusting*’, it was considered a more positive experience of evolving life changes:

…rather than coping. I always say adjusting… to the new situation.

(P08)

However, others thought the word ‘*coping*’ was relevant for the context it was being used in.

I think it's valid. Some people cope very well with ageing and others definitely do not. They're quite bitter and twisted about it. So yeah, coping, I think, is a good word.

(P07)

### 3.4 Relevance of Concepts Discussed in the CARE Intervention

The therapeutic components addressing loneliness and ageing are introduced in session 3. Within the patient resource a list of management strategies is provided, one of which is *“Reframing death as a learning experience increases acceptance”*. Most participants did not comprehend how death could be a learning experience and highlighted “reframing” was not an appropriate word to use in this context:

…if you go into death, it's the end. It doesn't mean you're going to learn anything from it.

(P04)

I don’t like reframing… you don’t have to justify why you’re gonna die prematurely.

(P25)

The concept of acquiring wisdom, discussed in session 4 was also perceived by participants as not reflecting how older Australians view wisdom. Participants disagreed with the assumption that being older means you're wiser:

I don't know that old means wise. A lot of people would disagree with that one.

(P19)

However, some participants perceived acquiring wisdom comes from the accumulation of knowledge and life experiences, asserting that wisdom is a product of living and learning over the years and that both experience and time have a role. For others passing on experiences rather than wisdom was a more acceptable way of framing this concept:

…the knowledge that you have from experience, you will turn that into wisdom when you have to…

(P03)

…you might not feel wiser, but you've got, a lifetime of experience, and you do draw on that and your friends' situations as well as your own.

(P19)

Discussions of ‘*wisdom*’ and ‘*keepers of meaning’ in* session 4 also explore death, dying and conversations about death. Participants highlighted the framing of this discussion felt ‘*religious’* (P18). The phrase ‘*living life in the face of death’* was not perceived as a commonly used expression in Australia, while one participant was concerned *‘doing it right, dying*’ would be interpreted locally as relating to voluntary assisted dying:

It almost slips into the religious side of dying rather than the mechanics of life.

(P06)

Well, to me, this [doing it right, dying’] is talking about where you can opt in to terminate your life. I feel uncomfortable about that.

(P23)

### 3.5 Clarity, Relevance and Understanding of Words and Images

Participants highlighted across session content many examples of words and phrases, that were not commonly used within Australia, or perceived as having a negative connotation, as well as images and examples that did not reflect Australian culture. For example, use of *‘infirmity’* rather than ‘*disability’.* Participants also noted that maintaining cognitive function is an important aspect of ageing and had a strong reaction to the use of ‘*Alzheimer's*’. This was considered confronting and participants suggested softening the language to a more general and less frightening term like dementia. Participants stressed the importance of clear, understandable language for participant engagement. For example, using complex words such as *‘amalgam’* instead of *‘mixture’* may discourage participants from engaging with the resources due to lack of understanding.

It’s not encouraging at all, because they’d be struggling with some of the words and the length of you know, it just needs to be simplified…

(P18)

When asked to reflect on specific aspects of the intervention content, participants perceived the session 1 introductory text explaining the background of the intervention and an overview of the programme, was overly complex and seemed to be aimed at healthcare professionals. Participants also highlighted that much of the data presented was US‐centric. Inclusion of simplified background information, with fewer acronyms and abbreviations, and Australian data was perceived as necessary to increase acceptability to Australian participants. Participants also noted the wording needed more kindness and compassion threaded through it.

As part of session 2, ageing and cancer are characterised as a ‘*double whammy’* and most participants took exception to this phrase, suggesting it was *“being quite flippant”* (P11) and one participant stated *“ageing isn't a double whammy, because you get there slowly. Cancer is.”* (P08). Similarly, use of the phrase “*when you get lemons, make lemonade”* was perceived as diminishing the significance of cancer and felt too *“American”* (P08).

When considering losses in later years of life an individual's career is currently referred to as their ‘*professional identity’.* Using ‘*working life’* was perceived as better reflecting the diversity of work experiences, although gender and generational views also influenced perceptions; for example, some men may define themselves by their job, while for others, feeling valued and contributing is more important:

…my last job was working in the local roadhouse …I retired from that job not a professional job.

(P08)

Similarly, current examples of social activities did not reflect the social lives of older Australians, particularly those in rural areas. For some, options like movies, concerts, and museums were less accessible due to physical limitations, financial constraints, and remote locations. Activities such as coffee catchups, barbecues, playing cards, and golf were seen as more appropriate and relevant. In rural areas, attending country races was a popular social activity and going to local clubs and sporting activities were also considered important for Australians.

…for us, it’d be catch up for coffee you know. It would be more Australian.

(P08)

While participants suggested updating the visual images incorporated into each session, one image in session 2 of a man in a wheelchair facing a window elicited a particularly strong response. Many felt it didn't resonate with their experience, and conveyed negativity as it was seen as sad, evoking fears of isolation and loneliness.

Well, the picture wouldn't relate to me at all, because it's someone in a wheelchair. So, they're probably in a nursing home…very few old people are in wheelchairs.

(P08)

Overall, the image was considered unrepresentative and not helpful in conveying a positive view of ageing and/or cancer. On the other hand, a picture of a couple sitting together laughing was received positively and was the image that participants chose to remain unchanged within the resources.

## 4 Discussion

Mental health interventions are more effective when they align with the cultural norms of country in which they are delivered [27]. This study highlighted that cultural adaptation of psycho‐oncology interventions is required, even between English‐speaking countries, to ensure cultural relevance and to maximise uptake. Adaptation frameworks such as FRAME [22] and the Cultural Adaptation Process Model [28] suggest that for successful adaptation the domains of language, context, persons, metaphors, concepts, goals, and methods need to be systematically addressed [28]. Addressing these domains strengthens the ecological validity and overall external validity of the adapted programme improving reach, engagement, and sustainability. This study sought to systematically review and adapt a US‐developed depression intervention for older people with cancer (CARE) to the Australian context.

Typically, language adaptation to intervention content is perceived to involve translation of intervention content from English to a second language. However, our results highlight the importance of ensuring that language reflects the linguistic characteristics of the country. For example, in this study, the term ‘*elders*’ holds significant cultural connotations for First Nations people in Australia and the term transcends age, embodying a deep sense of respect and recognition of cultural significance in First Nations communities [29]. While some participants were comfortable using this term to refer to older individuals more broadly, others suggested alternative terminology reflecting the respect for First Nations conceptualisations. Similarly, metaphors and examples are context specific as they reflect the values and customs within a culture. In our study, participants discussed differences in leisure activities examples in the resources and the lack of relevance to the Australian culture. Similarly, US words and phrases used such as “*when you get lemons, make lemonade”* were not commonly used in the Australian lexicon and highlighted as needing amendment. Additionally, participants felt the term ‘professional identity’ used in the resources was not inclusive for their culture and generation, as it excluded those with non‐professional jobs and has a gender bias, as historically, more men have held professional/managerial roles. If these subtle but important cultural adaptations are not addressed to ensure participants see themselves reflected in the content, interventions have been shown to not only have lower acceptability to potential participants but also are less effective overall [23].

In relation to the concepts within the intervention, the intertwining of ageism and racism weaved through the resources did not resonate with Australian participants. While ageism arguably exists in Australia and is often “so deeply ingrained in societal norms and values that it can be difficult to recognise” [30]. The comparison of ageism to racism within the resources did not resonate with participants, possibly due to a lack of experience of structural racism that could intersect with age‐related discrimination, making the comparison less relevant.

The CARE intervention is underpinned by the coping paradigm of Folkman and developmental stages of life [16] as outlined by Erikson and expanded by Vaillant [20]. Based on these frameworks, the intervention encompasses several core therapeutic techniques: reappraisal techniques important for successful ageing, and intervention techniques (coping strategies) shown to be effective with older adults. Overall participants perceived the intervention approach to be relevant, however conceptualisations of wisdom and death reappraisal did not resonate. Reframing is a cognitive process in which the interpretation of an experience is shifted to a more helpful frame [31]. However, reframing death, as outlined in the intervention content was contentious. Despite being older adults (> 70 years), participants did not feel their death was imminent, irrespective of their cancer diagnosis and lingering on the concept of death or changing the meaning of death to make it more positive did not seem valuable. The second concept, wisdom, is often associated with age, as it is grounded in accumulated life experience [32]. “Successful ageing” according to Erikson [33] is characterised by a sense of integrity, with better coping with age and illness, stronger ties to others, less depression and an acceptance of one's past life as it was lived [34]. These qualities are sometimes referred to as “wisdom” of our elders. Yet, consistent with previous literature, our participants highlighted not all older people are wise and wisdom may also diminish over time, as the ability to understand complex problems or regulate emotions in stressful situations, tend to decline with age [35], suggesting a need to define successful ageing in terms of life experience rather than wisdom per se. Similarly, the impact of life experience was also highlighted by some participants as influencing perceptions of coping, and coping strategies in particular.

Finally, in this study we explored the acceptability of the intervention methodology, an important factor that influences intervention implementation. While the overall utility of the intervention was confirmed, there was some ambivalence among participants about telephone delivery in an era of video conferencing in healthcare. Some participants feared a lack of non‐verbal cues would lessen rapport building, a significant concern for a psychological intervention. However, there is little evidence to support reduced therapeutic relationship, disclosure, empathy, attentiveness, or participation between telehealth modalities [36, 37]. The preferences reported by study participants likely reflect the eligibility criteria for this study including ability to use videoconferencing platforms and intervention feasibility must be considered in light of the digital divide between metropolitan and rural Australia, that has resulted in low telehealth uptake in rural Australia [38]. Therefore, it is essential to be able to deliver therapy for this group and be mindful that even in metropolitan centres, factors related to ageing or living with disability also impact on access, skills and confidence to use telehealth technologies [39].

## 5 Implications (Clinical and Research)

Adaptations are intentional modifications to maximise the appropriateness of an evidence‐based intervention to a new context. Cultural adaptation of interventions is crucial to provision of high‐quality and culturally responsive psycho‐oncology treatments. To do this we used a framework for cultural adaptation of evidence‐based psychological interventions to modify language and context without altering the core intervention content of an existing depression treatment programme tailored to the needs of older people with cancer. Some have argued that adaptations should be conducted only when there is evidence that the original intervention does not fit the populations' needs [40]. However, the results of our study highlight despite many similarities, even between English‐speaking countries, interventions need to be tailored to local context. Failure to take cultural differences into account can impact on acceptability and result in inefficient use of scarce resources and potentially cause harm. Adapting existing interventions is also more cost effective than developing new interventions, reducing the time from research to clinical implementation. In accordance with adaptation frameworks, the next step in our research is to trial the intervention with older Australians with cancer experiencing depression to confirm efficacy.

## 6 Limitations

The results of this study should be viewed in light of several limitations. Firstly, older people with cancer without internet access were excluded from the study. Despite this, when considering acceptability of telephone‐delivered interventions, participants acknowledged the benefits of telephone‐based intervention delivery for individuals with no internet access or technology literacy. Participants with insufficient English proficiency to read the provided resources or engage in qualitative interviews were also excluded. This may limit the generalisability of our findings, those who may be more educated or higher health literacy compared to the general Australian population may have participated. Similarly, the participants belonged to two homogenous cancer groups, blood and breast cancer, there could be variations in how different cancers impact patients. Furthermore, the predominantly white sample may account for the reduced impact of content comparing racism and ageism. Finally, as we were interested in the content of the intervention resources, participants were not required to have a diagnosis of depression, although 40% reported previously or currently seeking mental health support. Recruitment of older people with cancer and depression may have improved our understanding of acceptability but would have added additional challenges balancing need for treatment with intervention review and complicated the interview process.

## 7 Conclusion

While maintaining the core elements of the CARE intervention, we implemented key adaptations to ensure cultural appropriateness for older Australian cancer survivors. Cultural adaptation of psycho‐oncology interventions is crucial to enhance acceptability, reach, engagement, and sustainability. We integrated political alignment (e.g.*, Australian Cancer Plan* [41]*, Optimal Care Pathway for Older Adults with Cancer* [42]*),* economic accessibility (e.g., telephone delivery to overcome logistical and cost barriers), and cultural tailoring (e.g., language and values specific to older Australians with cancer) into CARE; demonstrating how these intersecting dimensions collectively inform acceptability and engagement.

## Author Contributions

**K.G.:** conceptualization, investigation, writing (original), formal analysis. **J.S.:** conceptualization, writing – review and editing, formal analysis. **H.D.:** conceptualization, writing – review and editing. **B.K.:** conceptualization, writing – review and editing. **C.N.:** conceptualization, writing – review and editing. **L.B.:** conceptualization, writing – review and editing. **M.K.:** conceptualization, writing – review and editing. **M.C.:** conceptualization, writing – review and editing. **A.C.:** writing – review and editing. **A.B.:** writing – review and editing. **S.H.:** investigation, writing – review and editing, formal analysis.

## Funding

The authors have nothing to report.

## Ethics Statement

Ethics approval for this study was provided by the University of Sydney HREC [2024/HE000898].

## Conflicts of Interest

The authors declare no conflicts of interest.

## Supporting information


Supporting Information S1

## Data Availability

The data that support the findings of this study are available on request from the corresponding author. The data are not publicly available due to privacy or ethical restrictions.
