# Supplementary material for: Cancer and Ageing Reflections for Elders (CARE): Australian Adaptation of a Psychotherapy for Older Adults With Cancer
Source: Psychooncology. 2026 Feb 14;35(2):e70402. doi: 10.1002/pon.70402 (PMC12906336; doi:10.1002/pon.70402)
Supplement: Supplementary file 1 — Supporting Information S1 [file PON-35-e70402-s001.docx]

**Supplementary Table 1: Summary of intervention content**

| Resource # | Resource/SESSION content |
| --- | --- |
| 1 | **CARE Study Background** - This resource explains why the study was needed, the research evidence behind it, the goals of the study and how the sessions with the mental health professional will work for patients who take part. It was expected a patient would read this ahead of their first session with the healthcare professional. |
| 1 | **Session One -** used in the first session between a patient and their healthcare professional. The healthcare professional will tell the patient about the purposes of the sessions and how they will run each week. The purpose of this first session is for the patient to “tell their story”, in their own words of what life has been like and the more recent history of changes with ageing, including, social changes, and cancer. The patient is asked to reflect and explain how these changes impact daily activities and socialising with others. |
| 2 | **Session Two -** used in the second session between a patient and their healthcare professional. Session two focuses on helping the patient with problem solving and that changing thoughts and ideas can lead to actual changes in behaviour. The patient will be encouraged to talk about any losses they have in their life, strategies used to manage them and any benefits that have come because of these losses. |
| 3 | **Session Three -** used in the third session between a patient and their healthcare professional. In this session the topic of loneliness is introduced, and the patient is asked to think about personal challenges, fears and worries associated with being alone more, and how this leads to loneliness and sadness and what they have used to cope with loneliness. The patient will be asked to reflect upon their experience of managing ageing in our society and to think about terms people use to talk about older people and what they think of these and how they make them feel. |
| 4 | **Session Four -** used in the fourth session between a patient and their healthcare professional. In this session the topic of wisdom and age is introduced, patients are asked to explore their own personal challenges and obstacles that get in the way of experiencing their own wisdom, value, and worth with other. They are asked to think about how they have changed as they have aged and since their cancer diagnosis, they will ask them how they have accepted some changes and to think about others that have been harder to accept and why. |
| 5 | **Session Five -** used in the fifth and final session between a patient and their healthcare professional. The patient would have had four sessions, prior to this one and will have worked through the topics of sessions 1-4 which will have included strategies for them to manage and cope with difficult situations they may encounter as part of growing older and experiencing cancer. This session the healthcare professional and patient will consider the previous sessions, and the patient will be asked to reflect upon the sessions, asking them what worked, what didn’t and what have they learnt. |
| 5 | **Booster Sessions -** there are four booster session, scheduled monthly after the completion of Session Five. The booster sessions are shorter – they are sessions for a patient to reflect on specific previous topics led by the healthcare professional. |

**Supplementary Table 2: Content analysis process**

| Step | Process |
| --- | --- |
| STep 1 | Researchers read, listen and familiarise themselves with each interview transcript and corresponding CARE intervention resource. |
| STEP 2 | ‘Big picture’ organising of the interview content and corresponding resource. An iterative process undertaken independently by researchers. Researchers identify words, phrases and examples that interviewees frequently commented or reflected on. |
| STEP 3 | Researchers bring together their ‘big picture’ coding and worked systematically through each resource, reviewing words and phrases identified. The specifics of what was being said by participants in the text was discussed and then labelled with a category that described this succinctly. |
| STEP 4 | Researchers refine categories formed and ensuring content appropriately categorised. Where consensus was mixed, concepts were reviewed by an independent member of the study research team initially and then consensus shared with the wider study advisory group to ensure decision agreement. |
| STEP 5 | Final interpretation and synthesis of the data using the content analysis codes developed was undertaken. The synthesis shows how the different content categories relate to each other and to the research question being explored and created a narrative for readers that explains the phenomenon. Changes to each resource content and the rationales were documented and are presented in content analysis summary tables. |
